# The relationship between quantitative human epidermal growth factor receptor 2 gene expression by the 21-gene reverse transcriptase polymerase chain reaction assay and adjuvant trastuzumab benefit in Alliance N9831

**DOI:** 10.1186/s13058-015-0643-7

**Published:** 2015-10-01

**Authors:** Edith A. Perez, Frederick L. Baehner, Steven M. Butler, E. Aubrey Thompson, Amylou C. Dueck, Farid Jamshidian, Diana Cherbavaz, Carl Yoshizawa, Steven Shak, Peter A. Kaufman, Nancy E. Davidson, Julie Gralow, Yan W. Asmann, Karla V. Ballman

**Affiliations:** Mayo Clinic, 4500 San Pablo Rd, Jacksonville, FL 32224 USA; Genomic Health, Inc, 301 Penobscot Drive, Redwood City, CA 94063 USA; Department of Health Sciences Research, University of California, 500 Parnassus Avenue, San Francisco, CA 94143 USA; Alliance Statistics and Data Center, Mayo Clinic, 13400 E. Shea Boulevard, Scottsdale, AZ USA; Norris Cotton Cancer Center, Dartmouth-Hitchcock Medical Center, 1 Medical Center Drive, Lebanon, NH 03766 USA; University of Pittsburgh Cancer Institute, 5150 Centre Avenue, Pittsburgh, PA 15232 USA; Seattle Cancer Care Alliance, 825 Eastlake Avenue East, Seattle, WA 98109 USA; Alliance Statistics and Data Center, 200 1st Street SW, Mayo Clinic, Rochester, MN 55905 USA

## Abstract

**Introduction:**

The N9831 trial demonstrated the efficacy of adjuvant trastuzumab for patients with human epidermal growth factor receptor 2 (HER2) locally positive tumors by protein or gene analysis. We used the 21-gene assay to examine the association of quantitative HER2 messenger RNA (mRNA) gene expression and benefit from trastuzumab.

**Methods:**

N9831 tested the addition of trastuzumab to chemotherapy in stage I–III HER2-positive breast cancer. For two of the arms of the trial, doxorubicin and cyclophosphamide followed by paclitaxel (AC-T) and doxorubicin and cyclophosphamide followed by paclitaxel and trastuzumab concurrent chemotherapy-trastuzumab (AC-TH), recurrence score (RS) and HER2 mRNA expression were determined by the 21-gene assay (Onco*type* DX®) (negative <10.7, equivocal 10.7 to <11.5, and positive ≥11.5 log_2_ expression units). Cox regression was used to assess the association of HER2 expression with trastuzumab benefit in preventing distant recurrence.

**Results:**

Median follow-up was 7.4 years. Of 1,940 total patients, 901 had consent and sufficient tissue. HER2 by reverse transcriptase polymerase chain reaction (RT-PCR) was negative in 130 (14 %), equivocal in 85 (9 %), and positive in 686 (76 %) patients. Concordance between HER2 assessments was 95 % for RT-PCR versus central immunohistochemistry (IHC) (>10 % positive cells = positive), 91 % for RT-PCR versus central fluorescence in situ hybridization (FISH) (≥2.0 = positive) and 94 % for central IHC versus central FISH. In the primary analysis, the association of HER2 expression by 21-gene assay with trastuzumab benefit was marginally nonsignificant (nonlinear *p* = 0.057). In hormone receptor-positive patients (local IHC) the association was significant (*p* = 0.002). The association was nonlinear with the greatest estimated benefit at lower and higher HER2 expression levels.

**Conclusions:**

Concordance among HER2 assessments by central IHC, FISH, and RT-PCR were similar and high. Association of HER2 mRNA expression with trastuzumab benefit as measured by time to distant recurrence was nonsignificant. A consistent benefit of trastuzumab irrespective of mHER2 levels was observed in patients with either IHC-positive or FISH-positive tumors. Trend for benefit was observed also for the small groups of patients with negative results by any or all of the central assays.

**Trial registration:**

Clinicaltrials.gov NCT00005970. Registered 5 July 2000.

**Electronic supplementary material:**

The online version of this article (doi:10.1186/s13058-015-0643-7) contains supplementary material, which is available to authorized users.

## Introduction

The human epidermal growth factor receptor 2 gene (HER2) has been reported to be amplified in 15–20 % of human breast cancers [1, 2]. HER2 gene amplification and protein overexpression are prognostic markers for aggressive disease and predictive markers for specific targeted therapies, including trastuzumab, pertuzumab, and lapatinib [3, 4]. Reliable testing methodology is critical and multiple discussions and publications related to this issue have been presented, addressing not only test reliability, and definition of “positivity,” but also which tests may best help predict the efficacy of anti-HER2 therapies for patients; however, the optimal target: protein, RNA, or DNA, and type of detection assay [immunohistochemistry (IHC), reverse transcriptase polymerase chain reaction (RT-PCR), or fluorescence in situ hybridization (FISH)] for HER2 remains controversial [5–16].

Persistent problems with test accuracy were recently highlighted by both the North Central Cancer Treatment Group (NCCTG) and National Surgical Adjuvant Breast and Bowel Project (NSABP) National Cancer Institute-supported Cancer Cooperative Groups who demonstrated that approximately 3–7 % of breast cancers formerly assessed as HER2 positive in local laboratories were called HER2-normal [IHC <10 % of cells with circumferential membrane staining; FISH HER2:centromere enumerator probe for chromosome 17 (CEP17) ratio <2.0] when evaluated centrally [12, 13, 17]. Interestingly, both studies demonstrated the efficacy of adjuvant trastuzumab added to chemoendocrine therapy not only for centrally assessed HER2-positive breast cancer but also for centrally assessed HER2-negative breast cancer [12, 13, 17, 18]. The reasons for the observed benefit in centrally assessed HER2-negative breast cancer may be due to a wide range of FISH and IHC methodologic variables including: differing methods of semiquantitation, differing cutoffs for positivity, discordance between pathologist interpretation and/or intratumoral heterogeneity. There is no firm evidence of a differential trastuzumab benefit due to quantitative differences in HER2 gene copy, messenger RNA (mRNA) expression or protein levels. In two large randomized trials a similar benefit of adding trastuzumab to adjuvant chemotherapy was observed for patients whose tumors were IHC 3+/FISH-negative or IHC 3+/FISH-positive; furthermore, no differential benefit was observed as a function of HER2 FISH ratio, HER2 copy number, or the presence of polysomy [13, 17, 19]. NSABP B-47 will ultimately illuminate whether the addition of trastuzumab to chemotherapy improves invasive disease-free survival (DFS) in women with resected node-positive or high-risk node-negative breast cancer which is reported as HER2-low by all HER2 testing performed. What are lacking in clinical practice today are highly quantitative, reproducible technology platforms for HER2 assessment that accurately select for benefit from anti-HER2 therapies.

The standardized RT-PCR platform used for the 21-gene assay is highly quantitative and reproducible. In fixed paraffin-embedded (FPE) tumor tissue its operational performance shows linearity over a >2000-fold RNA concentration range with an average accuracy of 0.3 %, coefficients of variation for the assay process are below 5.7 %, and assay variability contributed by instruments, operators, reagents and day-to-day variation are limited to less than 0.5 expression units (SD) [20, 21]. Since the discovery of HER2 multiple studies have compared mRNA expression by PCR with FISH and/or IHC [22–28]. Using 2007 American Society of Clinical Oncology/College of American Pathologists (ASCO/CAP) guidelines and excluding equivocal cases by both assays, a high degree of concordance (97 %) between HER2 mRNA levels by the Onco*type* DX assay and central laboratory FISH was observed in patients from the Kaiser study [22, 29]. A second HER2 concordance study between RT-PCR and central IHC in patients from ECOG E2197 also showed a high concordance (95 %) [23].

The role of quantitative RNA analysis of HER2 as predictor of benefit from trastuzumab has not been reported prior to the study reported here. This is a prospectively designed study to determine if quantitative levels of the ERBB2 gene expression level as assessed by mRNA coding for the HER2 protein, quantified by the 21-gene assay and reported as the HER2 single gene score, are predictive of the magnitude of benefit from the addition of trastuzumab to adjuvant chemotherapy in NCCTG (Alliance) N9831. The hypothesis was that increasing levels of expression are associated with increasing trastuzumab benefit. A secondary objective was to evaluate the concordance between HER2 mRNA expression level and protein assessment by IHC and gene copy number assessment by FISH.

## Methods

### Patients

The N9831 trial (the phase III trial of doxorubicin and cyclophosphamide followed by weekly paclitaxel with or without trastuzumab as adjuvant treatment for women with HER2-overexpressing or -amplified node-positive or high-risk node-negative breast cancer) was approved by participating institutional review boards (IRBs) [30]. The study had three arms: Arm A, doxorubicin and cyclophosphamide followed by weekly paclitaxel; Arm B, same as Arm A but followed by 1 year of sequential trastuzumab; Arm C, same as Arm A but with 1 year of concurrent trastuzumab, started the same day as paclitaxel. Results of the different arms of the N9831 trial were published in 2011, demonstrating that although each trastuzumab-containing arm led to statistically significant better DFS compared to chemotherapy alone, the largest difference was observed in the Arm C versus Arm A comparisons. The present analyses included only patients randomly assigned to Arms A or C, enrolled from May 25, 2000 through April 25, 2005, and tested for HER2 protein overexpression or gene amplification locally and at a central laboratory (Mayo Clinic, Rochester, MN, USA). All patients gave consent to participate.

### HER2 IHC and FISH testing methods

IHC staining was performed on paraffin-embedded 5-μm tissue sections using the HercepTest according to the manufacturer’s instructions (DAKO, Carpinteria, CA, USA) [12, 31, 32]. Assay control cell lines (SK-BR-3:3+, MDA-175:1+, MDA-231:0) provided on slides in the HercepTest kit were analyzed in each assay. Invasive carcinoma cells (and not benign epithelial or ductal carcinoma in situ cells) were used for the assessment of HER2 status of the tumor. Specimens were scored as per the instructions in the trastuzumab package insert. A specimen with at least 10 % invasive cells with complete membrane staining was classified as 3+ and considered HER2-positive according to pre-ASCO/CAP 2007 guidelines [29].

FISH analysis was performed on deparaffinized 5-μm tissue sections using the PathVysion ERBB2 DNA probe kit and the ERBB2/centromere 17(*HER2*/CEP17) probe mixture (Abbott Molecular, Des Plaines, IL, USA) [12, 31, 32]. For each case, a parallel hematoxylin and eosin (H&E)-stained slide was examined for regions of invasive carcinoma by a board-certified pathologist (D.W.V., R.P.K.). The completed tissue section was scanned by two certified cytogenetic technologists to detect any subpopulation of amplified cells. Thirty representative nuclei from the invasive tumor were scored by each technologist (60 nuclei total), with an overall evaluation performed by a board-certified pathologist (R.P.K., R.B.J.). When the red HER2 signals were clearly amplified (large clouds of amplification), we assigned ≥20 red signals and counted the green (CEP17) signals. For such cases, a number needs to be defined for the numerator and thus the ratio was defined as 20/average number of green signals per cell. As polysomy 17 (p17) increases, the ratio decreases. Scoring ranges were based on those determined for the US Food and Drug Administration-approved test for HER2 gene alterations in breast cancer (BC). A specimen with an HER2/CEP17 ratio ≥2.0 in invasive cells was classified as HER2 amplified and considered HER2 positive according to pre-ASCO/CAP 2007 guidelines [29].

Because many different HER2 and chromosome 17 alterations have been observed in BC, we independently categorized the HER2 FISH results on the basis of HER2 and CEP17 signal patterns [33–37]. For HER2-amplified tumors (HER2/CEP17 ratio ≥2), three ranges of CEP17 signals were observed: p17, ≥3 CEP17 signals in more than 30 % of nuclei; monosomy 17 (m17), 0 to 1 CEP17 signals in more than 60 % of nuclei; and normal (n17) all other cases. We carefully validated these polysomy and monosomy cutoffs by extensively analyzing our N9831 data and a large set (>10,000 cases) of clinical HER2 FISH assays concurrently performed by the central testing laboratory. Both cutoffs clearly distinguish chromosome 17 polysomic and monosomic cases from those cases without chromosome 17 centromere anomalies. All categorization thresholds were selected to reduce the rate of false-positive findings for gene amplification, gene deletion, and chromosome loss or gain. In our experience, these criteria have worked well to correct for truncation and nuclear overlap and the increase in four CEP17 signals due to G2M for nearly all solid tumors.

Quality control of the HER2 FISH test is assessed routinely according to standard College of American Pathologists and the American College of Medical Genetics guidelines. The performance of the assay as assessed on a monthly basis has been stable according to Westgard rules [38].

Eligibility criteria for N9831 trial enrollment were initially based on local laboratory HER2 test results (IHC score of 3+ or HER2/control probe ratio ≥2.0 or five or more gene copies of HER2) [12, 32]. After analysis of the first 119 specimens showed poor concordance between HER2 results from local and central (Mayo Clinic) laboratories, the protocol was amended (amendment 7), to require validation of HER2 positivity by the central laboratory for eligibility and study participation [32]. When the central laboratory’s IHC and FISH test results were both negative, the local site was contacted and another set of slides was submitted to a reference laboratory (Laboratory Corporation of America, Research Triangle Park, NC, USA). Enrollment into N9831 was then allowed only if HER2 positivity could be confirmed by IHC or FISH performed in the central or reference laboratories [12]. One hundred three patients with HER2-normal tumors (as shown by central laboratory IHC and FISH test results) continued in the trial because of local laboratory positivity (90 patients enrolled before amendment 7 was established) or because of reference laboratory positivity (13 patients enrolled after amendment 7). In the present analyses, HER2 status by IHC and FISH are based on the central laboratory tests originally done at Mayo Clinic after local laboratory testing being positive for HER2.

### HER2 mRNA testing methods

The Onco*type* DX breast cancer assay was performed as previously described for all available specimens with invasive carcinoma tissue (tumors were microdissected if there were less than 50 % carcinoma or if any contaminants were present, e.g., biopsy cavity elements or skin) [39]. All pathology was conducted blinded to clinical outcome. Microscopic tumor size was from pathology reports. Tumors were graded using the Nottingham system [40–42]. Tumors with less than 2.0 mm of invasive carcinoma were excluded. FPE tumor tissue was deparaffinized using Shandon xylene substitute followed by ethanol washes. RNA was extracted using Agencourt FormaPure kit (Beckman Coulter, Beverly, MA, USA), and treated with DNase I. All samples were confirmed free of genomic DNA by a β-actin-specific TaqMan® PCR assay. Total purified RNA content was quantified by Ribogreen® (Life Technologies, Carlsbad, CA, USA). Reverse transcription priming with random hexamers and gene-specific sequences was conducted using Omniscript RT kit (Qiagen, Valencia, CA, USA). TaqMan® PCR reactions were conducted in 384-well microtiter plates on ABI 7900HT (Applied Biosystems, Foster City, CA, USA) instruments. Gene expression was quantified by cycle threshold (C_T_) method, where expression was determined from the fractional number of cycles required to achieve a defined expression threshold. Gene expression was measured in triplicate, aggregated at the gene level, and normalized to the aggregate of five reference genes (ACTB, GAPDH, GUSB, RPLP0, and TFRC). Reference normalized expression ranged from 2 to 15 units where a 1-unit step corresponded to an approximate 2-fold change in RNA content. Prespecified analytical and quality metrics were applied as part of the rigorous, federally regulated Clinical Laboratory Improvement Amendments (CLIA) process. HER2 mRNA expression, incorporated into the 21-gene panel of the Onco*type* DX assay, was quantified, reference normalized, and reported as a single gene value. In accordance with CLIA requirements, HER2 expression range was qualified on each ABI 7900HT PCR unit using daily control standards corresponding to low and high gene expression levels, and data were verified prior to processing N9831 study samples. HER2 (ERBB2) expression levels corresponding to HER2 negative, equivocal, and HER2 positive were previously specified as <10.7, 10.7 to <11.5, ≥11.5, respectively. The cutoff for HER2 positivity by RT-PCR was initially identified in an exploratory analysis of 62 cases with IHC results (17 of which also had FISH results) [20]. This cutpoint was further examined in four studies that compared HER2 by RT-PCR with HER2 by IHC involving 78 cases [43], 249 cases [44], 45 cases [45], and 80 cases [46]. Based on these data, another cutpoint to establish an equivocal range was identified to align with the 2007 ASCO/CAP guidelines for HER2 testing, prespecified and validated in two subsequent positive clinical validation studies [22, 23].

### Study design and endpoints

The prespecified primary endpoint for the correlative quantitative gene analysis reported herein was distant recurrence-free interval (DRFI, i.e., time to distant recurrence), defined as the time (in years) from randomization to distant recurrence. The time to event for patients who died without distant recurrence was considered censored at the time of death, and local/regional recurrences were ignored. Disease-free survival (DFS) was a secondary endpoint, defined as the time from randomization to local, regional or distant recurrence, contralateral breast cancer including ductal carcinoma in situ, other second primary cancers, or death from any cause. DFS was censored at the last date that the patient was known to be to be DFS event-free.

The patients in this study were from either Arms A or C and had a successful Onco*type* DX assay, including both recurrence score (RS) and quantitative HER2 single gene results. All patients had both central IHC and FISH assessments as part of the parent N9831 study. Mayo Clinic IRB was the ethical body that approved our study.

### Statistical analysis

For the primary analysis, a multivariable Cox regression model, adjusting for nodal status (0, 1–3, 4–9, and 10+ positive nodes) as a main effect, was used to estimate the association between HER2 expression level and the benefit of trastuzumab for distant recurrence, comparing Arm C to Arm A. A natural cubic spline model with 2 degrees of freedom was used to allow for a potentially nonlinear association, and a likelihood ratio test was used to test for the significance of the ability to predict trastuzumab benefit. Statistical power was prospectively estimated at ≥90 % assuming there would be 88 and 47 evaluable patients with distant recurrences in Arms A and C (there were 102 and 45), respectively.

Additional Cox models were used to estimate the association between HER2 status by RT-PCR, IHC or FISH and the benefit of trastuzumab for distant recurrence. Kaplan-Meier estimates were generated for the proportion of patients free of distant recurrence as a function of time, and comparisons between groups were done using log-rank tests. Concordance of HER2 assessments was assessed according to the 2013 ASCO/CAP guidelines for HER2 testing [47]. All hypothesis tests were conducted at the 0.05 significance level, and two-sided *p* values and two-sided confidence intervals are reported.

The Onco*type* DX assay was performed by Genomic Health, while blinded to the N9831 study data, and statistical analyses were conducted jointly by the Alliance and Genomic Health, Inc. This study was approved by the North American Breast Cancer Group (NABCG) Correlative Sciences Committee. See the detailed statistical methods for further information (Additional file 1).

## Results

**Fig. 1:**
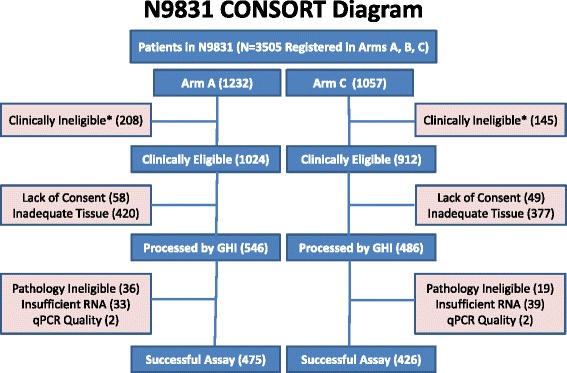
CONSORT diagram. *Reasons for clinical ineligibility: failed central and reference laboratory review for HER2 positivity (193), patient cancelled (22), patient lost to follow-up (96), and other reasons for clinical ineligibility (42), where the numbers are combined for Arms A and C. Note that Arm A includes 148 otherwise eligible patients who were enrolled during the Arm C closure

Of the 2289 patients registered to Arms A or C in the parent N9831 trial, 1936 were clinically eligible, and 1032 formalin FPE tumors were processed by Genomic Health, Inc., of which 901 (87.3 %) had successful Onco*type* DX assay results and were included in this analysis (Fig. 1). Of the 131 samples processed without successful assays, 55 (42.0 %) did not meet pathology criteria for the assay (insufficient or no tumor tissue or not primary tumor), 72 (55.0 %) had insufficient RNA, and 4 (3.1 %) did not meet RT-PCR quality metrics. Thus, 475 patients from Arm A and 426 from Arm C were analyzed for this study.

### Patient characteristics

Patient and tumor characteristics for the 901 patients included in this study are summarized in Table 1. The distributions of patient and tumor characteristics were compared between patients included in this study and the 1029 patients who were clinically eligible but not included in this study. There were no statistically significant differences, except for tumor size where, not unexpectedly, there was a slight shift toward larger tumor sizes among the patients included in this study relative to those not included (*p* = 0.03, Table 1). One hundred forty-seven distant recurrence events were observed during a median follow-up of 7.4 years.

**Table 1:** Patient and tumor characteristics: comparison or included versus not included clinically eligible patients

Characteristic	Clinically eligible and included (*n* = 901)	Clinically eligible but not included (*n* = 1029)	*p* value*
Arm assignment			
A	475 (53 %)	548 (53 %)	0.81
C	426 (47 %)	481 (47 %)	
Age at randomization (years)			
18–39	150 (17 %)	162 (16 %)	0.94
40–49	299 (33 %)	352 (34 %)	
50–59	292 (32 %)	344 (33 %)	
≥60	160 (18 %)	171 (17 %)	
Menopausal status			
Premenopausal	480 (53 %)	551 (54 %)	0.90
Postmenopausal	421 (47 %)	478 (46 %)	
Extent of surgery			
Breast sparing	354 (39 %)	396 (38 %)	0.72
Mastectomy	547 (61 %)	633 (62 %)	
Extent of nodal examination			
Axillary node dissection	816 (91 %)	925 (90 %)	0.62
Sentinel biopsy	85 (9 %)	104 (10 %)	
Histologically positive nodes			
0	125 (14 %)	132 (13 %)	0.82
1–3	424 (47 %)	497 (48 %)	
4–9	233 (26 %)	262 (25 %)	
≥10	119 (13 %)	138 (13 %)	
Tumor size (cm)			
≤2.0	340 (38 %)	429 (42 %)	0.03
2.1–4.9	481 (53 %)	532 (52 %)	
≥5	80 (9 %)	68 (7 %)	
Tumor grade			
1	16 (2 %)	12 (1 %)	1.00
2	233 (26 %)	274 (27 %)	
3	641 (71 %)	730 (71 %)	
Unknown	11 (1 %)	13 (1 %)	
Hormone receptor status			
ER or PR positive	481 (53 %)	552 (54 %)	0.89
ER and PR negative	420 (47 %)	476 (46 %)	
Unknown	-	1 (<1 %)	

*ER* estrogen receptor, *PR* progesterone receptor

*p value from chi-square test for nominal categories or Cochran-Mantel-Haenszel test for ordered categories

### Assessments of HER2 by IHC, FISH and RT-PCR

The pairwise concordance between HER2 mRNA expression level by RT-PCR, protein assessment by IHC, and gene copy number assessment by FISH, was assessed according to updated 2013 ASCO/CAP guidelines, in which equivocal assessments are classified with HER2-negative cases (Table 2). Among patients who were HER2 positive by local assessment and, after the early protocol amendment, by either central or reference assessment, overall concordance was high among the pairs of HER2 measures: 87.6 % between central IHC and RT-PCR, 84.4 % between central FISH and RT-PCR, and 85.8 % between central IHC and central FISH. The positive concordances of RT-PCR and central IHC (83.8 %) and of RT-PCR and central FISH (87.8 %) were comparable to that of central IHC and central FISH (88.9 %). The negative concordance of RT-PCR and central IHC (86.6 %) was similar to that of RT-PCR and central FISH (89.8 %), and greater than that of central IHC and central FISH (58.0 %). Among the 51 patients who were centrally negative by both IHC and FISH, 46 were 3+ by local IHC with no FISH result, and the remaining 5 were FISH-positive with no IHC result (ratios of 2.00, 2.15 and 2.20, with two missing ratio information). Concordance of HER2 status by RT-PCR and FISH, stratified by estrogen receptor (ER) status by IHC, showed a higher positive concordance in IHC ER-negative versus IHC ER-positive cases (88.5 % versus 78.6 %) (Additional file 2).

**Table 2:** Concordance of HER2 status by RT-PCR, IHC, and FISH

		HER2 status by RT-PCR				Concordance^a^	Positive concordance and negative concordance
		Negative	Equivocal	Positive	Total		
		*n* (%)	*n* (%)	*n* (%)	*n* (%)		
Central HER2 IHC	0	14 (1.6)	1 (0.1)	1 (0.1)	16 (1.8)	87.6 %	Pos. concordance = 87.8 %
	1+	19 (2.1)	6 (0.7)	0 (0.0)	25 (2.8)		Neg. concordance = 86.6 %
	2+	58 (6.5)	25 (2.8)	18 (2.0)	101 (11.2)		
							(Ref = IHC)
	3+	39 (4.3)	53 (5.9)	664 (73.9)	756 (84.2)		
	Total	130 (14.5)	85 (9.5)	683 (76.1)	898		
		HER2 status by RT-PCR				84.4 %	Pos. concordance = 83.8 %
		Negative	Equivocal	Positive	Total		
		*n* (%)	*n* (%)	*n* (%)	*n* (%)		Neg. concordance = 89.8 %
Central HER2 FISH ratio	<2	62 (7.0)	17 (1.9)	9 (1.0)	88 (10.0)		(Ref = FISH)
	≥2	64 (7.2)	65 (7.4)	667 (75.5)	796 (90.0)		
	Total	126 (14.3)	82 (9.3)	676 (76.5)	884		
		Central HER2 FISH ratio				85.8 %	Pos. concordance = 88.9 % (Ref = FISH)
		<2	≥2		Total		
		*n* (%)	*n* (%)		*n* (%)		
Central HER2 IHC	0	14 (1.6)	2 (0.2)		16 (1.8)		
	1+	14 (1.6)	10 (1.1)		24 (2.7)		Neg. concordance = 58.0 % (Ref = FISH)
	2+	23 (2.6)	76 (8.6)		99 (11.2)		
	3+	37 (4.2)	706 (80.0)		743 (84.2)		
	Total	88 (10.0)	794 (90.0)		882		

Positive concordance = (number of results positive by both methods)/(number of results positive by reference method)

Negative concordance = (number of results negative by both methods)/(number of results negative by reference method)

*HER2* human epidermal growth factor receptor 2, *RT-PCR* reverse transcriptase polymerase chain reaction, *IHC* immunohistochemistry, *FISH* fluorescence in situ hybridization

^a^Per 2013 ASCO/CAP guidelines, equivocal values are classified as HER2 negative for purposes of calculating concordance. Hence, IHC = 0, 1+ and 2+ are classified as HER2 negative and HER2 equivocal by RT-PCR is classified as HER2 negative

**Fig. 2:**
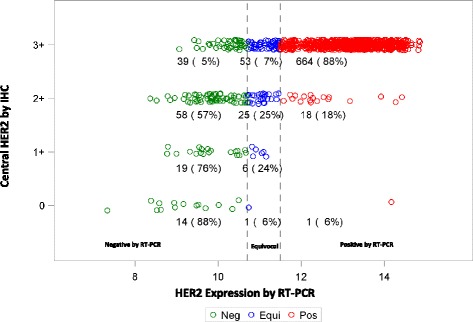
Distribution of HER2 by RT-PCR according to central HER2 by IHC. *HER2* human epidermal growth factor receptor 2, *IHC* immunohistochemistry, *RT-PCR* reverse transcriptase polymerase chain reaction

The association between the continuous HER2 mRNA expression by RT-PCR (log_2_ expression level) versus central HER2 by IHC is discernible (Spearman rank correlation = 0.54, 95 % CI 0.49–0.58), although there are broad ranges in HER2 expression level by RT-PCR within each IHC category (Fig. 2).

**Fig. 3:**
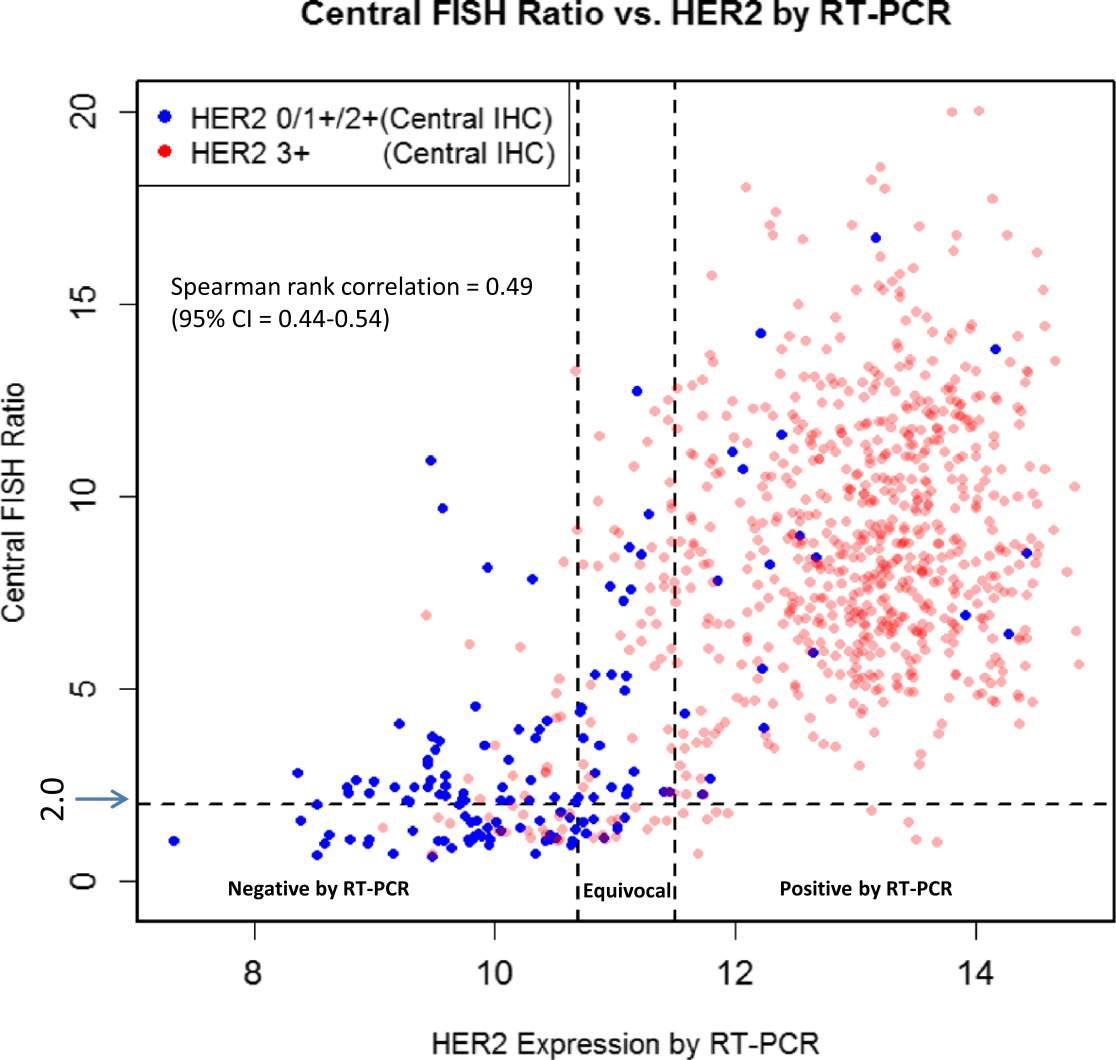
Distribution of HER2 by RT-PCR according to central FISH ratio. *FISH* fluorescence in situ hybridization, *HER2* human epidermal growth factor receptor 2, *RT-PCR* reverse transcriptase polymerase chain reaction

The association between continuous HER2 by RT-PCR and continuous central FISH ratio is depicted in Fig. 3. The Spearman rank correlation is 0.49 (95 % CI 0.44–0.54), similar to that between RT-PCR and central IHC.

Among the 882 patients with central IHC, FISH and RT-PCR assessments, all of whom were locally HER2 positive by IHC or FISH, 62 (7.0 %) cases were negative by both RT-PCR and central FISH, 25 of whom were also negative by central IHC.

### Prediction of trastuzumab benefit by assessment of HER2

**Fig. 4:**
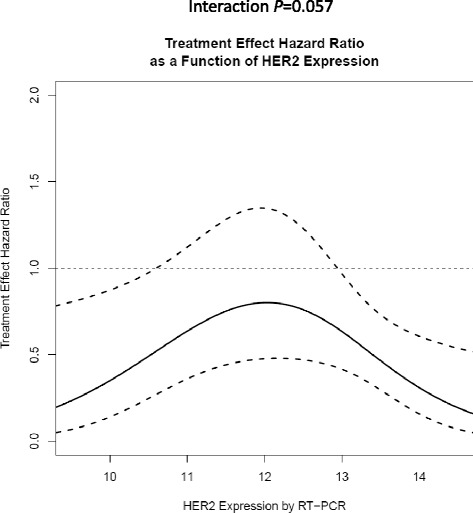
Hazard ratio for trastuzumab benefit as a continuous function of HER2 expression by RT-PCR. Estimate and 95 % confidence limits obtained from a Cox PH model for DRFI with a main effect for treatment arm (C versus A), a natural cubic spline for the main effect of HER2 by RT-PCR, a natural cubic spline for the interaction of HER2 by RT-PCR with treatment arm, and three indicator variables to adjust for nodal status (0, 1–3, 4–9 and 10+ positive nodes). *Solid line* = estimate of hazard ratio; *dashed lines* = lower and upper 95 % confidence limits. *DRFI* distant recurrence-free interval, *HER2* human epidermal growth factor receptor 2, *RT-PCR* reverse transcriptase polymerase chain reaction

In the primary analysis, continuous HER2 mRNA expression level by RT-PCR was not significantly associated with the magnitude of benefit from the addition of trastuzumab to adjuvant chemotherapy (nonlinear *p* = 0.057, Fig. 4). Hence, in this study of patients with locally HER2-positive tumors by IHC or FISH, and confirmed centrally in 94 % of patients, the hypothesis of increasing trastuzumab benefit with increasing HER2 mRNA expression was not supported.

**Fig. 5:**
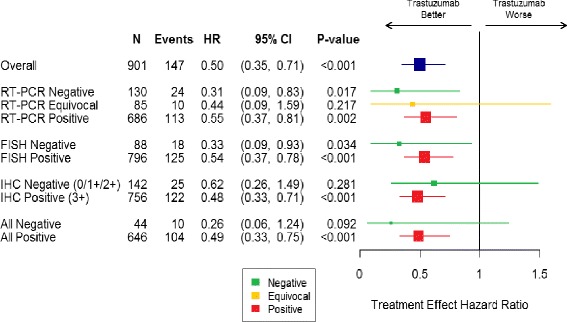
Trastuzumab benefit by HER2 status by RT-PCR, FISH and IHC. *FISH* fluorescence in situ hybridization, *HER2* human epidermal growth factor receptor 2, *IHC* immunohistochemistry, *RT-PCR* reverse transcriptase polymerase chain reaction

To further explore this observation, Cox proportional hazards models were used to estimate the treatment effect of trastuzumab, adjusted for nodal status (0, 1–3, 4–9 and 10+ positive nodes), overall and in different patient subsets. Hazard ratios for trastuzumab benefit are depicted in a forest plot, along with associated 95 % confidence intervals (Fig. 5). In all 901 patients, the addition of trastuzumab halved the risk of distant recurrence (hazard ratio = 0.50, *p* < 0.001). When stratified by HER2 status by RT-PCR, the hazard ratios for trastuzumab treatment were 0.31 (95 % CI 0.09–0.83) for HER2-negative, 0.44 (95 % CI 0.09–1.59) for HER2-equivocal, and 0.55 (95 % CI 0.37–0.81) for HER2-positive patients. There was a statistically significant benefit of trastuzumab in patients who were HER2 negative by RT-PCR, and in patients who were HER2 negative by central FISH. In the IHC-negative patients, and in the 44 patients who had negative central assessments of IHC, FISH and RT-PCR, there were nonsignificant hazard ratios toward benefit from trastuzumab. Of note, all of the 95 % confidence intervals for the hazard ratio for trastuzumab benefit overlapped with the overall hazard ratio of 0.50.

For secondary endpoint DFS, there also was no association between trastuzumab benefit and continuous HER2 by RT-PCR (*p* = 0.21). In patients who were HER2 negative by RT-PCR, the hazard ratio for trastuzumab treatment was 0.55 (95 % CI 0.26–1.12, *p* = 0.11). The hazard ratios for trastuzumab benefit were 0.81 (95 % CI 0.25–2.40) for HER2-equivocal, and 0.67 (95 % CI 0.49–0.90) for HER2-positive patients.

### Exploratory analyses of HER2 expression, hormone receptor status and prediction of trastuzumab benefit

Exploratory analyses were performed to evaluate the joint distribution of ER expression and HER2 expression, assess the effect of trastuzumab treatment by hormone receptor (HR) status, and the association between HER2 expression and trastuzumab benefit by HR status. A scatterplot of ER expression by RT-PCR versus HER2 expression by RT-PCR, stratified by ER status by local IHC (Additional file 3), indicates that these two measures are uncorrelated for tumors that are ER negative by IHC (Spearman rank correlation = -0.01, 95 % CI -0.10 to 0.09), but among tumors that were ER positive by local IHC, higher ER expression by RT-PCR was associated with lower HER2 expression by RT-PCR (Spearman correlation = -0.35, 95 % CI -0.43 to -0.27). However, a wide distribution of ER expression was seen regardless of HER2 expression level.

HR status was not a significant prognostic factor for DRFI in Cox models adjusted for nodal status, whether assessed by local IHC (*p* = 0.15) or RT-PCR (*p* = 0.86). Moreover, consistent with the N9831 parent trial results, a statistically significant trastuzumab benefit for distant recurrence was observed in both HR-positive and HR-negative subsets of patients, whether measured by local IHC or RT-PCR (Additional file 4), and no interaction between treatment and HR status was observed in Cox models adjusted for nodal status (*p* = 0.37 for HR status by local IHC and *p* = 0.18 for HR status by RT-PCR).

However, different results were obtained for DFS. HR status (positive versus negative) was a significant prognostic factor for DFS in Cox models adjusted for nodal status, whether assessed by local IHC (HR = 0.64, 95 % CI 0.49–0.83, *p* <0.001) or by RT-PCR (HR = 0.77, 95 % CI 0.59–0.997, *p* = 0.047). A statistically significant trastuzumab benefit for DFS was observed in HR-positive patients (*p* = 0.003 for local IHC and *p* <0.001 for RT-PCR) but not in the HR-negative subgroup (*p* = 0.13 and *p* = 0.36, respectively). There was no interaction between treatment and HR status by local IHC in a Cox model adjusted for nodal status (*p* = 0.24) but a similar model for HR status by RT-PCR indicated an interaction that did not quite achieve statistical significance (*p* = 0.051). Thus, HR status was neither prognostic nor predictive of trastuzumab benefit for DRFI, but prognostic with some evidence of differential trastuzumab benefit for DFS.

In addition, the association between HER2 expression level by RT-PCR and trastuzumab benefit was evaluated separately in the HR-positive and HR-negative patient subsets, using the same Cox model as in the primary analysis. In patients who were HR negative by local IHC, HER2 by RT-PCR was not associated with the magnitude of trastuzumab benefit for distant recurrence (nonlinear *p* = 0.69, Additional file 5). However, there was a significant association between HER2 by RT-PCR and trastuzumab benefit for distant recurrence in patients who were HR positive by local IHC (nonlinear *p* = 0.0015); the relationship was nonmonotonic, with large benefit at lower and higher expression levels, and no benefit for intermediate levels (Additional file 5). This pattern was explored further by examining Kaplan-Meier plots of time to distant recurrence comparing the two treatment arms by quartiles of HER2 expression by RT-PCR. In patients who were HR positive by local IHC, the greatest benefit from trastuzumab was observed in the lowest and highest quartiles of HER2 expression, with smaller benefit in the second and third quartiles (Additional file 6).

The same methods for the primary analysis were applied to continuous expression for each of the other 15 cancer-related genes in the Onco*type* DX assay, as well as the recurrence score, and none were statistically significant predictors of trastuzumab benefit.

## Discussion

The primary aim of this prospectively designed study was to determine if quantitative levels of the ERBB2 gene expression level as assessed by messenger RNA coding for the HER2 protein quantified by the 21-gene assay and reported as the HER2 single gene score are predictive of the magnitude of benefit from the addition of trastuzumab to adjuvant chemotherapy in NCCTG (Alliance) N9831. In this study of patients who were locally HER2 positive by IHC or FISH, continuous HER2 mRNA expression level by RT-PCR was not significantly associated with the magnitude of benefit from the addition of trastuzumab to adjuvant chemotherapy. Similarly, no association was observed for central IHC or central FISH ratio. Thus, the hypothesis of increasing trastuzumab benefit with increasing HER2 mRNA expression is not supported.

In all patients, the addition of trastuzumab approximately halved the risk of distant recurrence. This effect was noted both within RT-PCR HER2-positive and -negative patients. In particular, there was a statistically significant benefit of trastuzumab in patients who were HER2 negative by RT-PCR. Similarly, there was a statistically significant benefit of trastuzumab in patients who were HER2 negative by central FISH. Moreover, the 25 patients who had a local HER2-positive result (IHC or FISH), but negative central assessments of IHC, FISH and RT-PCR showed a smaller but nonsignificant hazard ratio toward greater benefit from trastuzumab. NSABP B-47 will determine whether the addition of trastuzumab to chemotherapy improves outcome in women with resected node-positive or high-risk node-negative breast cancer which is reported as HER2-low by all HER2 testing performed.

Statistically significant trastuzumab benefit was observed in both the HR-positive and HR-negative subsets of patients, whether measured by local IHC or RT-PCR [majority ER positive and/or progesterone receptor (PR) positive]. Among tumors that were ER positive by local IHC, higher ER expression by RT-PCR was associated with lower HER2 expression by RT-PCR, a relationship that is consistent with prior reports on protein expression, gene amplification status and gene expression [19, 48, 49]. Our data suggest a complex relationship between HER2 and ER as determinants of clinical benefit from trastuzumab added to adjuvant chemoendocrine therapy. There was a nonmonotonic association between HER2 mRNA and trastuzumab benefit in the HR-positive subgroup.

Surprisingly, the HER2 subgroup with the least clinical benefit from adjuvant trastuzumab actually had intermediately overexpressed—not the lowest—levels of HER2 mRNA, although the confidence intervals do not exclude a clinically important benefit of trastuzumab for these intermediate expression levels. This finding of a nonmonotonic interaction between HER2 expression and trastuzumab benefit in patients with the highest levels of ESR1-associated genes has been previously reported [50]. We cannot exclude clinical treatment benefit for any level of HER2 expression in this study. This finding is likely multifactorial and potential reasons for it include: that patients with tumors that express higher ER may have already derived maximum clinical benefit from antiestrogen therapy, that these tumors are biologically resistant to trastuzumab, or there is a complex interaction between hormone therapy, chemotherapy and trastuzumab benefit and tumor biology. These findings have prompted further studies using advanced molecular techniques to determine if other genes or gene groups will be better predictors of trastuzumab benefit [51].

The secondary aim of this study was to assess concordance among the three central assays. Overall concordance, assessed based on the current ASCO/CAP guidelines, was high for RT-PCR versus IHC (87.6 %) and FISH (84.4 %) and similar to the concordance observed between central IHC and central FISH (85.8 %) [10]. The concordance was higher and comparable to the previously reported concordance studies when calculated using the 2007 ASCO/CAP guidelines [22, 23, 29]. The positive concordances of RT-PCR and central IHC and of RT-PCR and central FISH were comparable to that of central IHC and central FISH, whereas the negative concordance of RT-PCR and central IHC was similar to that of RT-PCR and central FISH, but greater than that of central IHC and central FISH. Although all patients were locally HER2 positive by IHC or FISH, the clinical significance of these HER2 concordance results in this randomized clinical trial population, N9831, is that they are consistent with, and therefore supportive of, the high degree of concordance of HER2 assessment by RT-PCR with central IHC and FISH assays from prior randomized clinical trial and cohort populations that are more representative for HER2 testing [22, 23]. Although the concordance was high for classification into positive and negative categories, the associations between the quantitative HER2 mRNA expression by RT-PCR and semiquantitative central IHC and FISH measures was modest and there were broad ranges in HER2 expression level by RT-PCR within each IHC/FISH category. The causes of this variability are likely multifactorial. True biologic differences between RNA levels and DNA gene amplification offer one possibility [5]. Sources of analytic variability are another: not all FISH assays are the same nor are all RT-PCR assays [52]. With respect to RT-PCR technology, differences in RNA extraction methods, reverse transcription, PCR protocols, instruments, primer/probe selection and reagent manufacturing can contribute to significant assay variation. The Onco*type* DX assay uses controls, calibrators, reference ranges (for quantitative single gene ER, PR and HER2 results) and utilizes normalization to address differences in RNA quality [20, 53–55]. The variability (SD) contributed by instruments, operators, reagents and day-to-day variation for RT-PCR using Onco*type* DX is less than 0.5 expression units [20, 21]. Sources of preanalytic variability (e.g., delay to fixation, choice of fixative or duration of fixation) may also play a role and their impact in HER2 assessment by FISH is well described [56, 57].

Our HER2 concordance results are not consistent with those of Dabbs et al. [58]. Three institutions reported on a predominantly ER-positive HER2-negative convenience sample of 843 total cases: 36 (4 %) HER2 positive, 23 (3 %) equivocal and 784 (93 %) centrally HER2 negative [58, 59]. Of the 784 HER2-negative patient cases, 779 (99 %) were classified as negative by RT-PCR while of the 36 IHC/FISH-positive cases, by RT-PCR only 10 (28 %) were reported as positive, 12 (33 %) as equivocal, and 14 (39 %) as negative. In only one of the three participating institutions from that study, did the investigators retest the discordant cases, e.g., those cases that had been IHC/FISH positive but RT-PCR negative (N = 9), in which the repeat test used the same FPE block for all assays, IHC/FISH and RT-PCR. When the same block sent for RT-PCR testing was retested using FISH, 44 % (4/9) were converted from positive to equivocal. It is not clear if these analyses were done in a blinded manner, whether limited foci of amplification were used to call positivity, or if these differences are due to heterogeneity. Tumor cell dilution has also been suggested as a source of discordance; however, we assessed the percentage of tumor cells and the tumor area and, consistent with the observation of Christgen et al., there were no significant differences between concordant and discordant cases [25]. The discordances between FISH and IHC, and RT-PCR and IHC and FISH again highlight that there are differences between HER2 assays and their targets. These differences, and other factors including differences in HER2 concordance as a function of ER status, further demonstrate why definitive studies need to ideally be done in large randomized trial populations with appropriate HER2 distributions and clinical outcomes in order to minimize sources of potential bias [10, 25, 60, 61].

The strengths of this study include the large landmark N9831 randomized trial population of patients with HER2-positive breast cancer, randomized to chemotherapy ± trastuzumab treatment, using high-quality central and reference laboratories with standardized methods for FISH and RT-PCR assays [20, 21, 52, 62]. There are also some limitations to consider when interpreting these study results. All patients had tumors that were deemed to be HER2 positive using protein or gene analysis by local laboratories, and our findings may not be generalizable to the broader patient population. Also, our study included only 47 % of the clinically eligible patients from the parent study, although patient characteristics (except for tumor size) were similar between patients included and not included.

## Conclusions

In summary, our data do not show that trastuzumab benefit increases with increasing HER2 mRNA expression. They do support the high concordance with central IHC and FISH assays as previously reported. We do not recommend HER2 testing by RT-PCR replacing IHC or FISH assays in standard practice.

## Additional files

Additional file 1:
**Supplemental detailed statistical methods.** (DOCX 13 kb) Additional file 2: Table S1. Supplemental table of the concordance of HER2 status by RT-PCR and FISH, stratified by ER status by IHC. (DOCX 19 kb) Additional file 3: Figure S1. Scatterplot of ER expression by RT-PCR and HER2 expression by RT-PCR. Colors indicate ER status by local IHC: *Blue* = ER-positive, *Red* = ER-negative. (PDF 189 kb) Additional file 4: Figure S2. Kaplan-Meier plots of distant recurrence by treatment arm, by hormone receptor status (local IHC and RT-PCR). (PDF 274 kb) Additional file 5: Figure S3. Hazard ratio for trastuzumab benefit as a continuous function of HER2 expression by RT-PCR, by HR status by local IHC. Estimate and 95 % confidence limits obtained from a Cox PH model for DRFI with a main effect for treatment arm (C versus A), a natural cubic spline for the main effect of HER2 by RT-PCR, a natural cubic spline for the interaction of HER2 by RT-PCR with treatment arm, and three indicator variables to adjust for nodal status (0, 1–3, 4–9 and 10+ positive nodes). Solid line = estimate of hazard ratio; dashed lines = lower and upper 95 % confidence limits. (PDF 177 kb) Additional file 6: Figure S4. Kaplan-Meier plots of distant recurrence by treatment arm for patients who are HR positive by local IHC, by quartile of HER2 expression by RT-PCR. (PDF 263 kb)

## Abbreviations

ASCO/CAP: American Society of Clinical Oncology/College of American Pathologists
BC: breast cancer
CEP17: centromere enumerator probe for chromosome 17
CLIA: Clinical Laboratory Improvement Amendments
CT: cycle threshold
DFS: disease-free survival
DRFI: distant recurrence-free interval
ER: estrogen receptor
FISH: fluorescence in situ hybridization
FPE: fixed paraffin-embedded
H&E: hematoxylin and eosin
HER2: human epidermal growth factor receptor 2
HR: hormone receptor
IHC: immunohistochemistry
IRB: institutional review board
m17: monosomy 17
mRNA: messenger RNA
n17: normal
NABCG: North American Breast Cancer Group
NCCTG: North Central Cancer Treatment Group
NSABP: National Surgical Adjuvant Breast and Bowel Project
p17: polysomy 17
PR: progesterone receptor
RS: recurrence score
RT-PCR: reverse transcriptase polymerase chain reaction

## References

[1] Slamon DJ, Clark GM, Wong SG, Levin WJ, Ullrich A, McGuire WL (1987). Human breast cancer: correlation of relapse and survival with amplification of the HER-2/neu oncogene. Science..

[2] Chia S, Norris B, Speers C, Cheang M, Gilks B, Gown AM (2008). Human epidermal growth factor receptor 2 overexpression as a prognostic factor in a large tissue microarray series of node-negative breast cancers. J Clin Oncol..

[3] Slamon DJ, Leyland-Jones B, Shak S, Fuchs H, Paton V, Bajamonde A (2001). Use of chemotherapy plus a monoclonal antibody against HER2 for metastatic breast cancer that overexpresses HER2. N Engl J Med..

[4] Geyer CE, Forster J, Lindquist D, Chan S, Romieu CG, Pienkowski T (2006). Lapatinib plus capecitabine for HER2-positive advanced breast cancer. N Engl J Med..

[5] Sauter G, Lee J, Bartlett JM, Slamon DJ, Press MF (2009). Guidelines for human epidermal growth factor receptor 2 testing: biologic and methodologic considerations. J Clinical Oncol..

[6] Ahmed SS, Iqbal J, Thike AA, Lim AS, Lim TH, Tien SL (2011). HER2/neu revisited: quality and interpretive issues. J Clin Pathol..

[7] De P, Smith BR, Leyland-Jones B (2010). Human epidermal growth factor receptor 2 testing: where are we?. J Clin Oncol..

[8] Vogel UF (2010). Confirmation of a low HER2 positivity rate of breast carcinomas - limitations of immunohistochemistry and in situ hybridization. Diagn Pathol..

[9] Ross JS (2009). Breast cancer biomarkers and HER2 testing after 10 years of anti-HER2 therapy. Drug News Perspect..

[10] Wolff AC, Hammond ME, Hicks DG, Dowsett M, McShane LM, Allison KH (2014). Recommendations for human epidermal growth factor receptor 2 testing in breast cancer: American Society of Clinical Oncology/College of American Pathologists clinical practice guideline update. Arch Pathol Lab Med..

[11] Shah SS, Ketterling RP, Goetz MP, Ingle JN, Reynolds CA, Perez EA (2010). Impact of American Society of Clinical Oncology/College of American Pathologists guideline recommendations on HER2 interpretation in breast cancer. Hum Pathol..

[12] Perez EA, Suman VJ, Davidson NE, Martino S, Kaufman PA, Lingle WL (2006). HER2 testing by local, central, and reference laboratories in specimens from the North Central Cancer Treatment Group N9831 intergroup adjuvant trial. J Clin Oncol..

[13] Perez EA, Reinholz MM, Hillman DW, Tenner KS, Schroeder MJ, Davidson NE (2010). HER2 and chromosome 17 effect on patient outcome in the N9831 adjuvant trastuzumab trial. J Clin Oncol..

[14] Press MF, Finn RS, Cameron D, Di Leo A, Geyer CE, Villalobos IE (2008). HER-2 gene amplification, HER-2 and epidermal growth factor receptor mRNA and protein expression, and lapatinib efficacy in women with metastatic breast cancer. Clin Cancer Res..

[15] Press MF, Sauter G, Bernstein L, Villalobos IE, Mirlacher M, Zhou JY (2005). Diagnostic evaluation of HER-2 as a molecular target: an assessment of accuracy and reproducibility of laboratory testing in large, prospective, randomized clinical trials. Clin Cancer Res..

[16] Press MF, Slamon DJ, Flom KJ, Park J, Zhou JY, Bernstein L (2002). Evaluation of HER-2/neu gene amplification and overexpression: comparison of frequently used assay methods in a molecularly characterized cohort of breast cancer specimens. J Clin Oncol..

[17] Paik S, Kim C, Wolmark N (2008). HER2 status and benefit from adjuvant trastuzumab in breast cancer. N Engl J Med..

[18] Paik S, Kim C, Jeong J, Geyer CE, Romond E, Mejia-Mejia O (2007). Benefit from adjuvant trastuzumab may not be confined to patients with IHC 3+ and/or FISH-positive tumors: Central testing results from NSABP B-31. J Clin Oncol..

[19] Dowsett M, Procter M, McCaskill-Stevens W, de Azambuja E, Dafni U, Rueschoff J (2009). Disease-free survival according to degree of HER2 amplification for patients treated with adjuvant chemotherapy with or without 1 year of trastuzumab: the HERA Trial. J Clin Oncol..

[20] Cronin M, Pho M, Dutta D, Stephans JC, Shak S, Kiefer MC (2004). Measurement of gene expression in archival paraffin-embedded tissues: development and performance of a 92-gene reverse transcriptase-polymerase chain reaction assay. Am J Pathol..

[21] Cronin M, Sangli C, Liu ML, Pho M, Dutta D, Nguyen A (2007). Analytical validation of the Oncotype DX genomic diagnostic test for recurrence prognosis and therapeutic response prediction in node-negative, estrogen receptor-positive breast cancer. Clin Chem..

[22] Baehner FL, Achacoso N, Maddala T, Shak S, Quesenberry CP, Goldstein LC (2010). Human epidermal growth factor receptor 2 assessment in a case-control study: comparison of fluorescence in situ hybridization and quantitative reverse transcription polymerase chain reaction performed by central laboratories. J Clin Oncol..

[23] Badve S, Gray R, Childs BH, Maddala T, Liu ML, Rowley S (2008). HER2 concordance between central laboratory immunohistochemistry and quantitative reverse transcription polymerase chain reaction in intergroup trial E2197.

[24] Lehmann-Che J, Amira-Bouhidel F, Turpin E, Antoine M, Soliman H, Legres L (2011). Immunohistochemical and molecular analyses of HER2 status in breast cancers are highly concordant and complementary approaches. Br J Cancer..

[25] Christgen M, Harbeck N, Gluz O, Nitz U, Kreipe HH (2012). Recognition and handling of discordant negative human epidermal growth factor receptor 2 classification by Oncotype DX in patients with breast cancer. J Clin Oncol..

[26] Park S, Wang HY, Kim S, Ahn S, Lee D, Cho Y (2014). Quantitative RT-PCR assay of HER2 mRNA expression in formalin-fixed and paraffin-embedded breast cancer tissues. Int J Clin Exp Pathol..

[27] Di Fiore PP, Pierce JH, Kraus MH, Segatto O, King CR, Aaronson SA (1987). erbB-2 is a potent oncogene when overexpressed in NIH/3T3 cells. Science.

[28] Coussens L, Yang-Feng TL, Liao YC, Chen E, Gray A, McGrath J (1985). Tyrosine kinase receptor with extensive homology to EGF receptor shares chromosomal location with neu oncogene. Science..

[29] Wolff AC, Hammond ME, Schwartz JN, Hagerty KL, Allred DC, Cote RJ (2007). American Society of Clinical Oncology/College of American Pathologists guideline recommendations for human epidermal growth factor receptor 2 testing in breast cancer. J Clin Oncol..

[30] Perez EA, Suman VJ, Davidson NE, Gralow JR, Kaufman PA, Visscher DW (2011). Sequential versus concurrent trastuzumab in adjuvant chemotherapy for breast cancer. J Clin Oncol..

[31] Perez EA, Roche PC, Jenkins RB, Reynolds CA, Halling KC, Ingle JN (2002). HER2 testing in patients with breast cancer: poor correlation between weak positivity by immunohistochemistry and gene amplification by fluorescence in situ hybridization. Mayo Clin Proc..

[32] Roche PC, Suman VJ, Jenkins RB, Davidson NE, Martino S, Kaufman PA (2002). Concordance between local and central laboratory HER2 testing in the breast intergroup trial N9831. J Natl Cancer Inst..

[33] Bose S, Mohammed M, Shintaku P, Rao PN (2001). Her-2/neu gene amplification in low to moderately expressing breast cancers: possible role of chromosome 17/Her-2/neu polysomy. Breast J..

[34] Morrison LE, Jewell SS, Usha L, Blondin BA, Rao RD, Tabesh B (2007). Effects of ERBB2 amplicon size and genomic alterations of chromosomes 1, 3, and 10 on patient response to trastuzumab in metastatic breast cancer. Genes Chromosomes Cancer..

[35] Reinholz MM, Bruzek AK, Visscher DW, Lingle WL, Schroeder MJ, Perez EA (2009). Breast cancer and aneusomy 17: implications for carcinogenesis and therapeutic response. Lancet Oncol..

[36] Vanden Bempt I, Drijkoningen M, De Wolf-Peeters C (2007). The complexity of genotypic alterations underlying HER2-positive breast cancer: an explanation for its clinical heterogeneity. Curr Opin Oncol..

[37] Zaczek A, Welnicka-Jaskiewicz M, Bielawski KP, Jaskiewicz J, Badzio A, Olszewski W (2008). Gene copy numbers of HER family in breast cancer. J Cancer Res Clin Oncol..

[38] Westgard JO, Barry PL, Hunt MR, Groth T (1981). A multi-rule Shewhart chart for quality control in clinical chemistry. Clin Chem..

[39] Paik S, Shak S, Tang G, Kim C, Baker J, Cronin M (2004). A multigene assay to predict recurrence of tamoxifen-treated, node-negative breast cancer. N Engl J Med..

[40] Elston CW, Ellis IO (1991). Pathological prognostic factors in breast cancer. I. The value of histological grade in breast cancer: experience from a large study with long-term follow-up. Histopathology.

[41] Elston CW, Ellis IO (1999). The breast, vol. 13. 3rd ed.

[42] Tavassoli FA, Devilee P (2003). World Health Organization Classification of Tumours. Pathology and Genetics of Tumours of the Breast and Female Genital Organs.

[43] Cobleigh MA, Tabesh B, Bitterman P, Baker J, Cronin M, Liu ML (2005). Tumor gene expression and prognosis in breast cancer patients with 10 or more positive lymph nodes. Clin Cancer Res..

[44] Esteva FJ, Sahin AA, Cristofanilli M, Coombes K, Lee SJ, Baker J (2005). Prognostic role of a multigene reverse transcriptase-PCR assay in patients with node-negative breast cancer not receiving adjuvant systemic therapy. Clin Cancer Res..

[45] Mina L, Soule SE, Badve S, Baehner FL, Baker J, Cronin M (2007). Predicting response to primary chemotherapy: gene expression profiling of paraffin-embedded core biopsy tissue. Breast Cancer Res Treat..

[46] Chang JC, Makris A, Gutierrez MC, Hilsenbeck SG, Hackett JR, Jeong J (2008). Gene expression patterns in formalin-fixed, paraffin-embedded core biopsies predict docetaxel chemosensitivity in breast cancer patients. Breast Cancer Res Treat..

[47] Wolff AC, Hammond ME, Hicks DG, Dowsett M, McShane LM, Allison KH (2013). Recommendations for human epidermal growth factor receptor 2 testing in breast cancer: American Society of Clinical Oncology/College of American Pathologists clinical practice guideline update. J Clin Oncol..

[48] Konecny G, Pauletti G, Pegram M, Untch M, Dandekar S, Aguilar Z (2003). Quantitative association between HER-2/neu and steroid hormone receptors in hormone receptor-positive primary breast cancer. J Natl Cancer Inst..

[49] Wirapati P, Sotiriou C, Kunkel S, Farmer P, Pradervand S, Haibe-Kains B (2008). Meta-analysis of gene expression profiles in breast cancer: toward a unified understanding of breast cancer subtyping and prognosis signatures. Breast Cancer Res..

[50] Pogue-Geile KL, Kim C, Jeong JH, Tanaka N, Bandos H, Gavin PG (2013). Predicting degree of benefit from adjuvant trastuzumab in NSABP trial B-31. J Natl Cancer Inst..

[51] Perez EA, Thompson EA, Ballman KV, Anderson SK, Asmann YW, Kalari KR (2015). Genomic analysis reveals that immune function genes are strongly linked to clinical outcome in the North Central Cancer Treatment Group n9831 adjuvant trastuzumab trial. J Clin Oncol..

[52] Gown AM, Goldstein LC, Barry TS, Kussick SJ, Kandalaft PL, Kim PM (2008). High concordance between immunohistochemistry and fluorescence in situ hybridization testing for HER2 status in breast cancer requires a normalized IHC scoring system. Mod Pathol..

[53] Ming Z, Bronner M, Magi-Galluzzi C, Tuthhill R, Baehner FL, Liu ML (2007). Optimized RNA extraction and RT-PCR assays provide successful molecular analysis on a wide variety of archival fixed tissues.

[54] Vandesompele J, De Preter K, Pattyn F, Poppe B, Van Roy N, De Paepe A, et al. Accurate normalization of real-time quantitative RT-PCR data by geometric averaging of multiple internal control genes. Genome Biol. 2002;3:RESEARCH0034. 10.1186/gb-2002-3-7-research0034 PMC126239 12184808

[55] Suzuki T, Higgins PJ, Crawford DR (2000). Control selection for RNA quantitation. Biotechniques..

[56] Tapia C, Schraml P, Simon R, Al-Kuraya KS, Maurer R, Mirlacher M (2004). HER2 analysis in breast cancer: reduced immunoreactivity in FISH non-informative cancer biopsies. Int J Oncol..

[57] Khoury T, Sait S, Hwang H, Chandrasekhar R, Wilding G, Tan D (2009). Delay to formalin fixation effect on breast biomarkers. Mod Pathol..

[58] Dabbs DJ, Klein ME, Mohsin SK, Tubbs RR, Shuai Y, Bhargava R (2011). High false-negative rate of HER2 quantitative reverse transcription polymerase chain reaction of the Oncotype DX test: an independent quality assurance study. J Clin Oncol..

[59] Bartlett JM, Starczynski J (2011). Quantitative reverse transcriptase polymerase chain reaction and the Oncotype DX test for assessment of human epidermal growth factor receptor 2 status: time to reflect again?. J Clin Oncol..

[60] Park MM, Ebel JJ, Zhao W, Zynger DL (2014). ER and PR immunohistochemistry and HER2 FISH versus oncotype DX: implications for breast cancer treatment. Breast J..

[61] Dvorak L, Dolan M, Fink J, Varghese L, Henriksen J, Gulbahce HE (2013). Correlation between HER2 determined by fluorescence in situ hybridization and reverse transcription-polymerase chain reaction of the oncotype DX test. Appl Immunohistochem Mol Morphol.

[62] Yaziji H, Goldstein LC, Barry TS, Werling R, Hwang H, Ellis GK (2004). HER-2 testing in breast cancer using parallel tissue-based methods. JAMA..

