# Supplementary material for: The relationship between quantitative human epidermal growth factor receptor 2 gene expression by the 21-gene reverse transcriptase polymerase chain reaction assay and adjuvant trastuzumab benefit in Alliance N9831
Source: Breast Cancer Res. 2015 Oct 1;17:133. doi: 10.1186/s13058-015-0643-7 (PMC4589954; doi:10.1186/s13058-015-0643-7)
Supplement: Additional file 1: — Supplemental detailed statistical methods. (DOCX 13 kb) [file 13058_2015_643_MOESM1_ESM.docx]

**Additional file 1**

**Detailed Statistical Methods**

The primary objective was to determine if HER2 gene expression as measured by the Onco*type* DX breast cancer assay was predictive of the magnitude of benefit from the addition of trastuzumab to adjuvant chemotherapy with regard to time to distant recurrence. The working hypothesis was that there would be no trastuzumab benefit below a threshold value of HER2 expression, and that beyond that threshold benefit would increase monotonically with increasing levels of expression. To accommodate this potentially non-linear association, natural cubic splines with 2 degrees of freedom were used with knots at the minimum, median, and maximum HER2 score. For the primary analysis, a Cox proportional hazards regression model was fit to time to distant recurrence, with a main effect for treatment arm (C vs. A), a natural cubic spline for the main effect of HER2 by RT-PCR, a natural cubic spline for the interaction of HER2 by RT-PCR with treatment arm, and 3 indicator variables to adjust for nodal status (0, 1-3, 4-9 and 10+ positive nodes). The primary hypothesis test was a likelihood ratio test with 2 degrees of freedom, comparing the full model specified above with the reduced model that excluded the interaction effects. Similar analyses were performed for HER2 by central FISH and by central IHC.

Statistical power for the primary hypothesis was calculated assuming that the distribution of HER2 scores was approximately normally distributed, that there was no trastuzumab benefit below a HER2 score of 10.7 (the cutpoint between HER2-negative and HER2-equivocal), and that the log hazard ratio for trastuzumab benefit increased linearly with increasing HER2 expression above 10.7.
