# Supplementary material for: The relationship between quantitative human epidermal growth factor receptor 2 gene expression by the 21-gene reverse transcriptase polymerase chain reaction assay and adjuvant trastuzumab benefit in Alliance N9831
Source: Breast Cancer Res. 2015 Oct 1;17:133. doi: 10.1186/s13058-015-0643-7 (PMC4589954; doi:10.1186/s13058-015-0643-7)
Supplement: Additional file 2: Table S1. — Supplemental table of the concordance of HER2 status by RT-PCR and FISH, stratified by ER status by IHC. (DOCX 19 kb) [file 13058_2015_643_MOESM2_ESM.docx]

Table S1: Concordance of HER2 Status by RT-PCR and FISH, Stratified by ER Status by IHC

| **ER-Positive by IHC** | | **HER2 Status by RT-PCR** | | |  | **Concordance*** | **Positive Concordance & Negative Concordance** |
| --- | --- | --- | --- | --- | --- | --- | --- |
|  |  | **Negative**  **n (%)** | **Equivocal**  **n (%)** | **Positive**  **n (%)** | **Total**  **n (%)** |  |  |
| **Central HER2 FISH Ratio** | **<2** | 45 (10.2) | 12 (2.7) | 4 (0.9) | 61 (13.9) | 80.7% | Pos. Concordance = 78.6%  Neg. Concordance = 93.4% |
|  | **≥2** | 39 (8.9) | 42 (9.5) | 298 (67.7) | 379 (86.1) |  |  |
|  | **Total** | 84 (19.1) | 54 (12.3) | 302 (68.6) | 440 |  |  |
|  |  |  | | |  |  |  |
| **ER-Negative by IHC** | | **HER2 Status by RT-PCR** | | |  | 88.1% | Pos. Concordance = 88.5%  Neg. Concordance = 81.5% |
|  |  | **Negative**  **n (%)** | **Equivocal**  **n (%)** | **Positive**  **n (%)** | **Total**  **n (%)** |  |  |
| **Central HER2 FISH Ratio** | **<2** | 17 (3.8) | 5 (1.1) | 5 (1.1) | 27 (6.1) |  |  |
|  | **≥2** | 25 (5.6) | 23 (5.2) | 369 (83.1) | 417 (93.9) |  |  |
|  | **Total** | 42 (9.5) | 28 (6.3) | 374 (84.2) | 444 |  |  |

* Per 2013 ASCO/CAP Guidelines, equivocal values are classified as HER2-negative for purposes of calculating concordance. Hence, HER2-equivocal by RT-PCR is classified as HER2-negative.

Positive Concordance = (Number of results positive by both methods)/(Number of results positive by FISH)

Negative Concordance = (Number of results negative by both methods)/(Number of results negative by FISH)
