# Supplementary figures and images for: The relationship between quantitative human epidermal growth factor receptor 2 gene expression by the 21-gene reverse transcriptase polymerase chain reaction assay and adjuvant trastuzumab benefit in Alliance N9831
Source: Breast Cancer Res. 2015 Oct 1;17:133. doi: 10.1186/s13058-015-0643-7 (PMC4589954; doi:10.1186/s13058-015-0643-7)

Figure S1

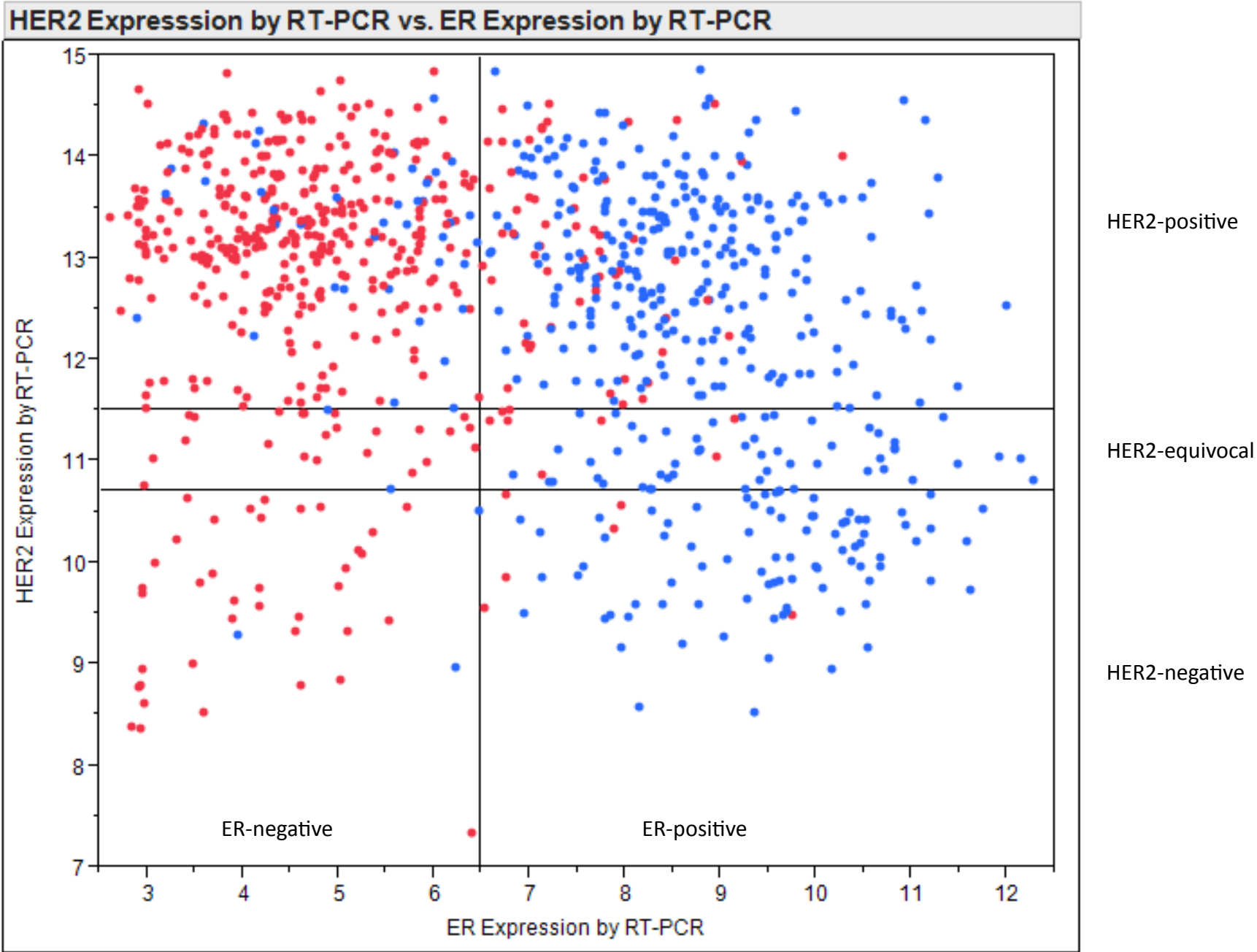

Supplement: Additional file 3: Figure S1. — Scatterplot of ER expression by RT-PCR and HER2 expression by RT-PCR. Colors indicate ER status by local IHC: Blue = ER-positive, Red = ER-negative. (PDF 189 kb) [file 13058_2015_643_MOESM3_ESM.pdf]

Figure S2

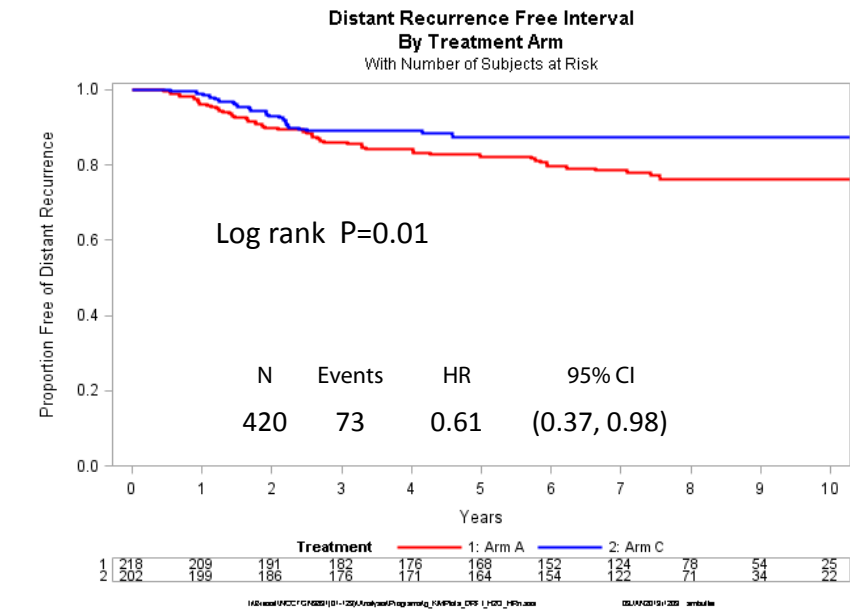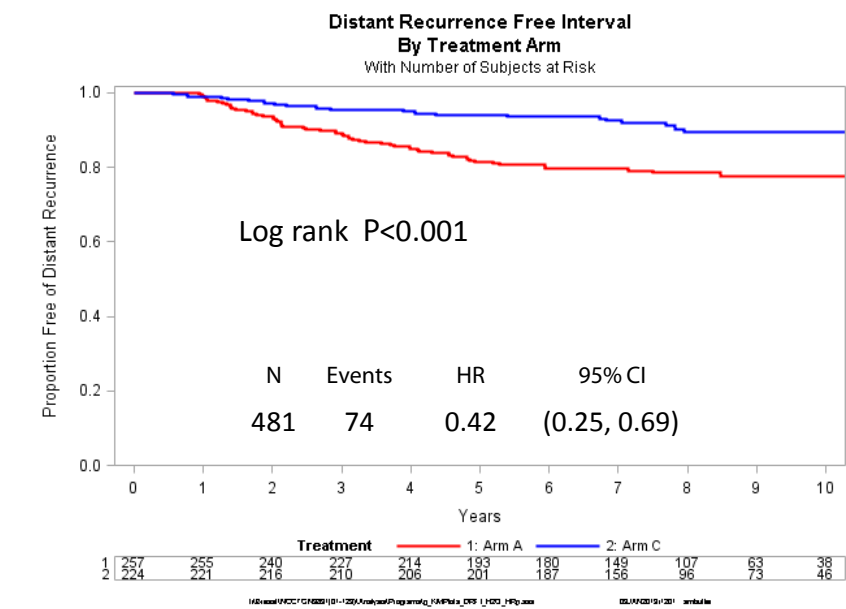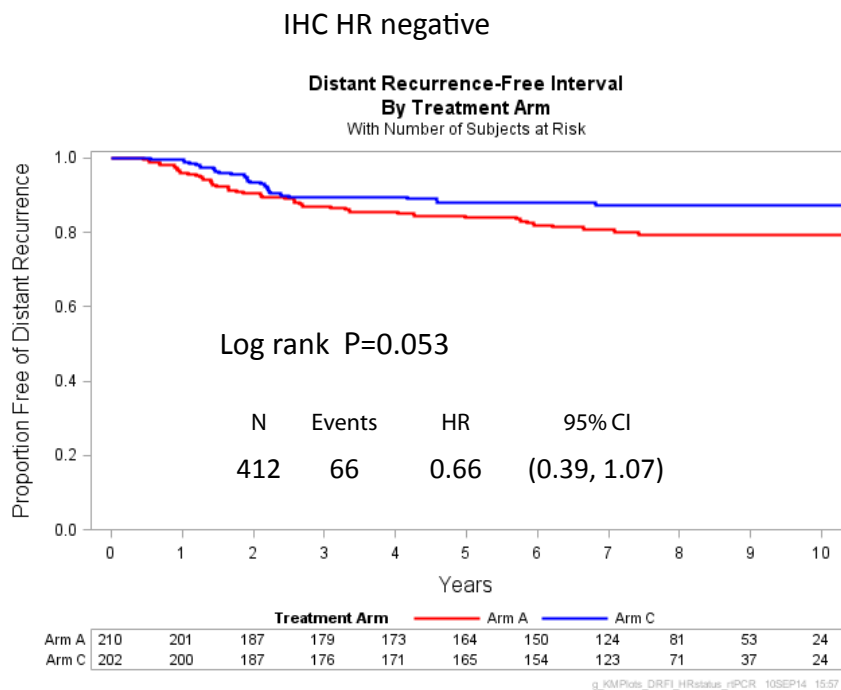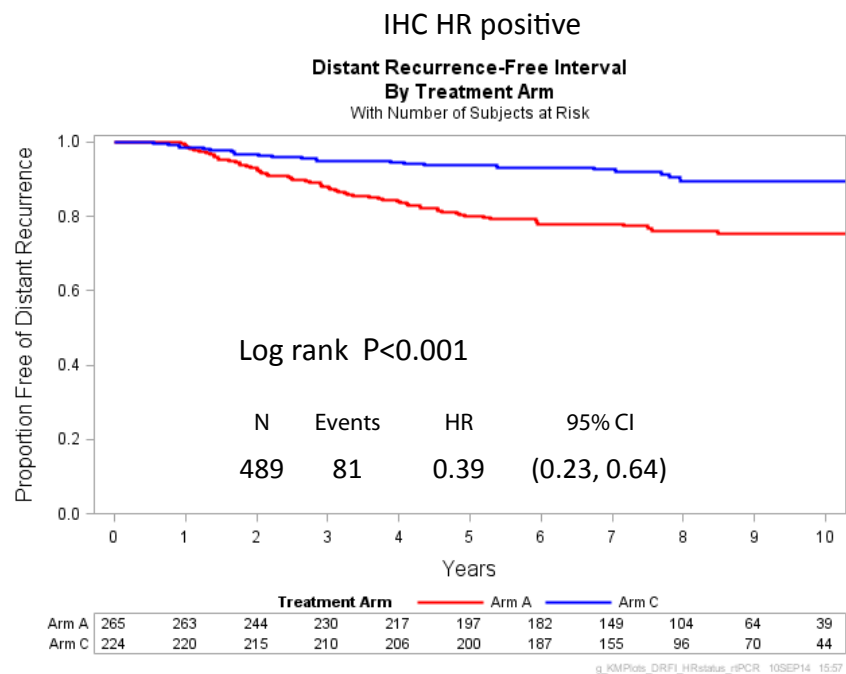

RT-PCR HR negative

RT-PCR HR positive

Supplement: Additional file 4: Figure S2. — Kaplan-Meier plots of distant recurrence by treatment arm, by hormone receptor status (local IHC and RT-PCR). (PDF 274 kb) [file 13058_2015_643_MOESM4_ESM.pdf]

Figure S4

# KM Plots by HER2 Quartile: HR Positive by Local IHC

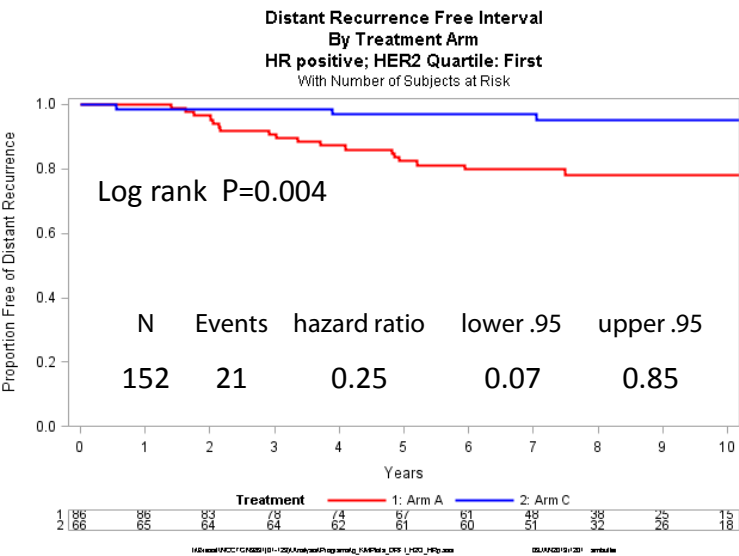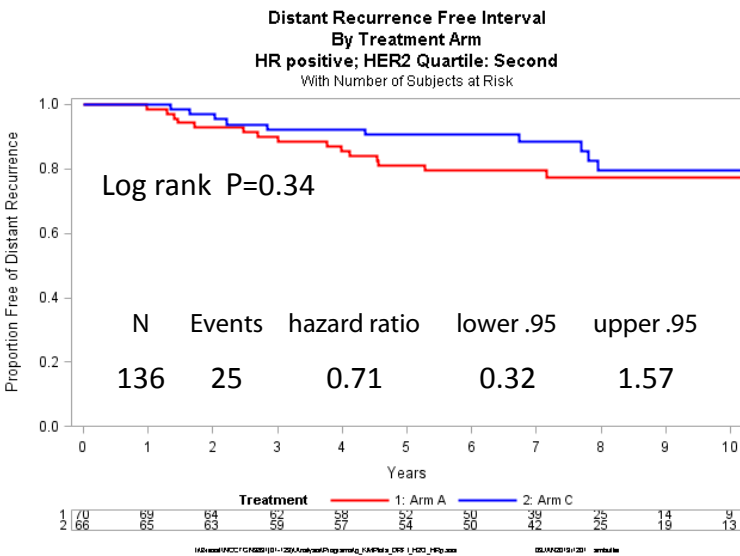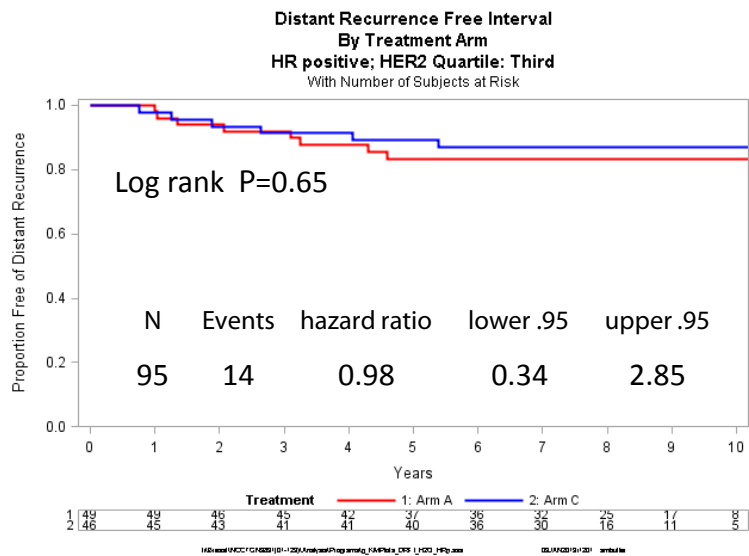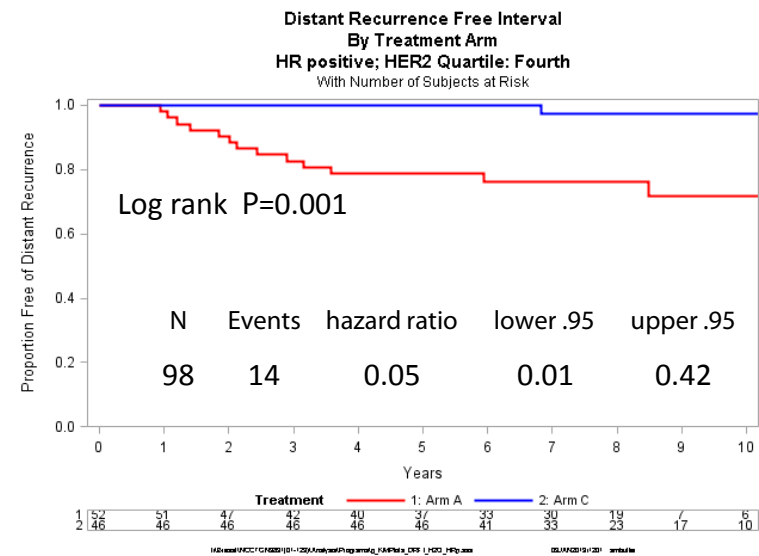

Supplement: Additional file 6: Figure S4. — Kaplan-Meier plots of distant recurrence by treatment arm for patients who are HR positive by local IHC, by quartile of HER2 expression by RT-PCR. (PDF 263 kb) [file 13058_2015_643_MOESM6_ESM.pdf]
