# Supplementary material for: The relationship between quantitative human epidermal growth factor receptor 2 gene expression by the 21-gene reverse transcriptase polymerase chain reaction assay and adjuvant trastuzumab benefit in Alliance N9831
Source: Breast Cancer Res. 2015 Oct 1;17:133. doi: 10.1186/s13058-015-0643-7 (PMC4589954; doi:10.1186/s13058-015-0643-7)

Figure S3

Treatment Effect Hazard Ratio as a Function of HER2 by HR Status by Local IHC

Interaction P=0.0015

HR positive

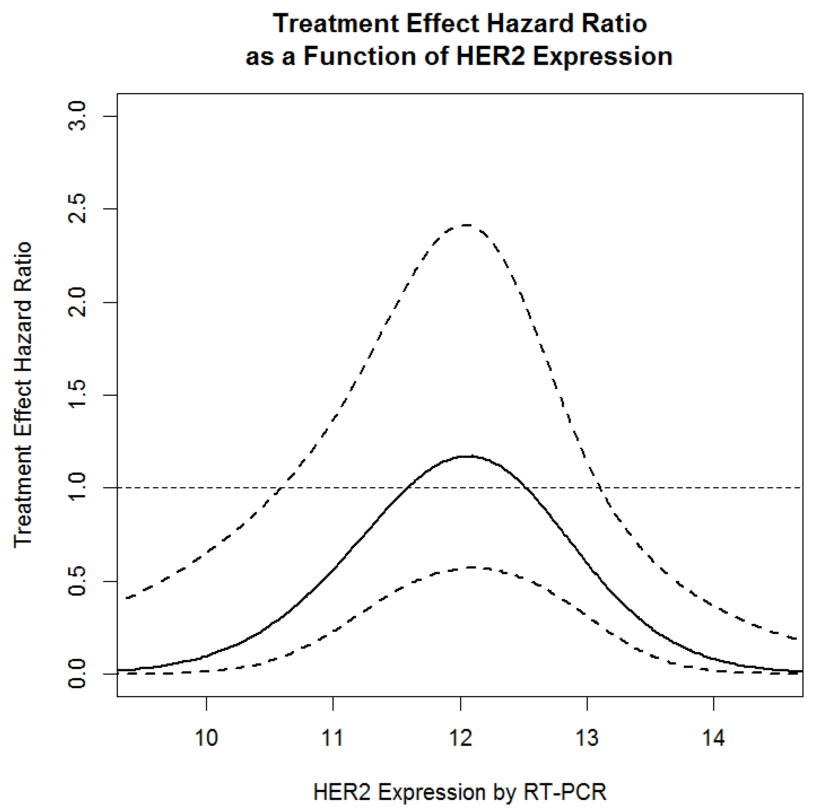

Interaction P=0.69

HR negative

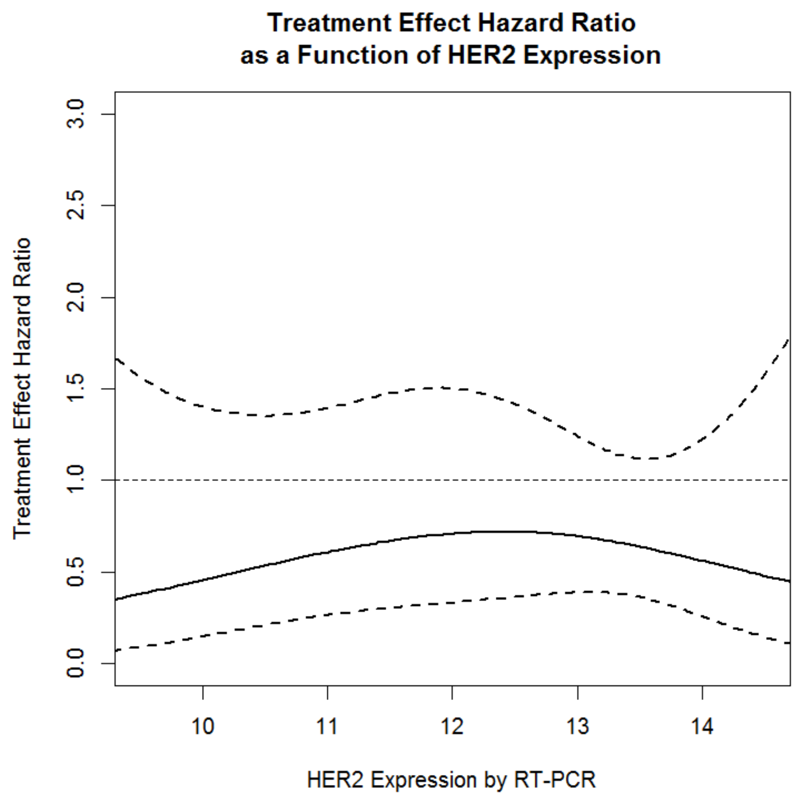

Supplement: Additional file 5: Figure S3. — Hazard ratio for trastuzumab benefit as a continuous function of HER2 expression by RT-PCR, by HR status by local IHC. Estimate and 95 % confidence limits obtained from a Cox PH model for DRFI with a main effect for treatment arm (C versus A), a natural cubic spline for the main effect of HER2 by RT-PCR, a natural cubic spline for the interaction of HER2 by RT-PCR with treatment arm, and three indicator variables to adjust for nodal status (0, 1–3, 4–9 and 10+ positive nodes). Solid line = estimate of hazard ratio; dashed lines = lower and upper 95 % confidence limits. (PDF 177 kb) [file 13058_2015_643_MOESM5_ESM.pdf]
